# Loss of copy of *MIR1-2* increases *CDK4* expression in ileal neuroendocrine tumors

**DOI:** 10.1038/s41389-020-0221-4

**Published:** 2020-03-20

**Authors:** Tanupriya Contractor, Chris R. Harris

**Affiliations:** 1 Raymond and Beverly Sackler Foundation, New Brunswick, NJ USA; 2 Department of Surgery, Rutgers Robert Wood Johnson Medical School, New Brunswick, NJ USA; 3 Rutgers Cancer Institute of New Jersey, New Brunswick, NJ USA

**Keywords:** Cancer genetics, Endocrine cancer

## Abstract

Ileal neuroendocrine tumors (I-NETs) are the most common tumors of the small intestine. Although I-NETs are known for a lack of recurrently mutated genes, a majority of tumors do show loss of one copy of chromosome 18. Among the genes on chromosome 18 is *MIR1-2*, which encodes a microRNA, MIR1-3p, with high complementarity to the mRNA of *CDK4*. Here we show that transfection of neuroendocrine cell lines with MIR1-3p lowered CDK4 expression and activity, and arrested growth at the G1 stage of the cell cycle. Loss of copy of *MIR1-2* in ileal neuroendocrine tumors associated with increased expression of *CDK4*. Genetic events that attenuated RB activity, including loss of copy of *MIR1-2* as well as loss of copy of *CDKN1B* and *CDKN2A*, were more frequent in tumors from patients with metastatic I-NETs. These data suggest that inhibitors of CDK4/CDK6 may benefit patients whose I-NETs show loss of copy of *MIR1-2*, particularly patients with metastatic disease.

## Introduction

Neuroendocrine cells are found throughout the body, from the pituitary to the rectum. To prevent hormonal imbalances, these hormone-producing cells are usually under tight growth regulation. But as with many cell types, growth controls can go awry, allowing hyperplasias or even tumors to form. Because neuroendocrine tumors (NETs) often remain well-differentiated and continue to produce hormones, the first symptoms experienced by patients with NETs are often hormone-related. For instance, insulinomas can cause hypoglycemia, while parathyroid tumors can cause frequent kidney stones by altering calcium metabolism.

One of the most common sites of NETs is the ileum of the small intestine^1–3^. Ileal neuroendocrine tumors (I-NETs) are usually multifocal, well-differentiated tumors that produce the hormone serotonin. Overproduction of serotonin can lead to chronic diarrhea and skin flushing; these symptoms are part of a condition known as carcinoid syndrome. Carcinoid syndrome is associated with liver metastasis and poor outcome^4^. Notably, the incidence of carcinoid syndrome is increasing^5^. In all, there are about 2500 new cases of I-NETs per year in the United States^1,2^, making them the most common tumors of the small intestine. A majority of I-NETs are already metastatic by the time of diagnosis. Half of all patients diagnosed with metastatic I-NETs do not survive beyond 5 years^1^.

**Fig. 1:**
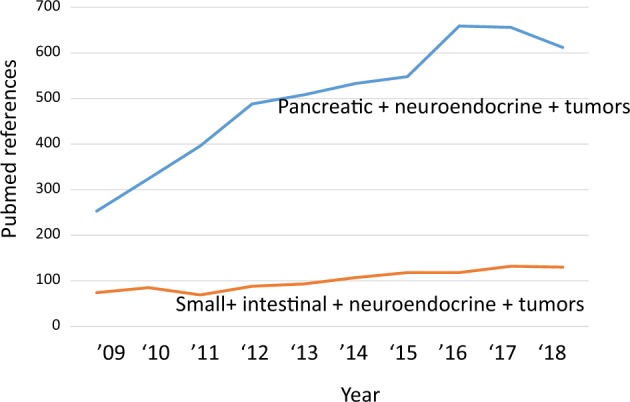
A comparison of research interest in ileal and pancreatic neuroendocrine tumors. This graph shows, by year, the number of Pubmed abstracts referring to the search terms “small + intestinal + neuroendocrine + tumors” (lower line) or to the search terms “pancreatic + neuroendocrine + tumors” (upper line).

In the clinic, I-NETs are as common as pancreatic neuroendocrine tumors (PNETs)^2^, yet are much less studied. For instance, in Fig. 1, there is a comparison of the number of published abstracts that mention “small intestinal neuroendocrine tumors” with the number of abstracts that mention “pancreatic neuroendocrine tumors.” Part of this research disparity is due to a lack of research tools for I-NETs. I-NET cell lines have been published^6–8^, but have not been made publicly available. There are also multiple mouse models for PNETs^9–14^, whereas until very recently^15^ I-NETs had not been found in mice. I-NETs also grow slowly, are difficult to detect clinically, and have proven nearly impossible to xenograft into mice.

Another difference that likely fuels the disparity in published work on PNETs and I-NETs is the fact that PNETs have recurrent mutations in genes of great research interest, such as the chromatin remodeling genes *MEN1*, *DAXX*, and *ATRX*^16^, whereas the genetic drivers of I-NETs have long remained a mystery^15^. I-NETs lack mutations in the genes associated with many cancers, such as *TP53*, *RB1*, *BRCA1*, *MYC*, *AKT1*, or *PIK3CA*. I-NETs are also one of the few types of NETs that are not observed in patients with familial neuroendocrine tumor syndromes, and thus are not caused by alleles of *MEN1* or *MEN2* that produce these syndromes^17–19^. In certain families, there is evidence for a predisposition to I-NETs, which links to genes other than *MEN1* or *MEN2*^20–22^. Overall there is a low frequency of mutation in I-NETs, more similar to the low frequency of mutation found in pediatric cancers than to the higher frequency of mutations in most of the tumor types that arise in older patients^23^. The only gene recurrently mutated in I-NETs is *CDKN1B*, which is altered in only 5–8% of I-NETs^23–25^; however, I-NETs have not been reported in mice with *CdkN1b* mutations.

The only common genetic change in I-NETs is the complete or near-complete loss of one copy of chromosome 18, which is found in well over half of patient samples^26–28^. While this suggests that chromosome 18 harbors a tumor suppressor gene that prevents I-NETs from forming, no specific gene on chromosome 18 has ever been linked to I-NETs. In this study, we show that a gene on chromosome 18, *MIR1-2*, produces a microRNA that can block expression of CDK4 and thereby activate the RB1 tumor suppressor protein, resulting in cell cycle arrest of neuroendocrine cell lines. These data suggest that chromosome 18 loss triggers I-NETs by dysregulating the RB pathway and activating the cell cycle. These data suggest that patients with I-NETs may benefit from CDK4/CDK6 inhibitors.

## Results

Because of the high incidence of chromosome 18 loss in I-NETs, we examined this chromosome for the presence of potential tumor suppressor genes. We focused on potential activators of the RB1 tumor suppressor, because the RB1 pathway is associated with several types of NETs^29–31^ and also because this pathway can be attenuated by loss of *CDKN1B*, which is the only gene that is recurrently mutated in I-NETs^23,25,32^. None of the well-studied activators of RB1, such as the CDK inhibitors *CDKN1A*, *CDKN1B*, *CDKN2A*, and *CDKN2B*, reside on chromosome 18, but we did identify a potential RB1 activator by searching for microRNAs with complementarity to negative regulators of the RB1 pathway. Using TargetScan 7.2 software (http://www.targetscan.org/vert_72/), we searched for broadly conserved miRNAs with 7mer-A1, 7mer-M8, or 8mer complementarity^33^ to the mRNAs of *CDK4*, *CDK6*, *CCND1*, *CCND2*, and *CCND3*. We then used the UCSC Genome Browser (http://genome.ucsc.edu/) to match these microRNAs to the chromosomes of the genes encoding them within human genome release GRCh38. Out of 105 microRNA genes identified by this approach, only one, *MIR1-2*, resided on chromosome 18 (Supplementary Table 1). *MIR1-2* encodes MIR1-3p, which is complementary to sequences within the untranslated regions (UTRs) of mRNAs for *CDK4*, *CDK6*, *CCND1*, and *CCND2*. Interestingly, decreased MIR1-3p expression has previously been detected in patients with advanced I-NETs^34,35^.

**Fig. 2:**
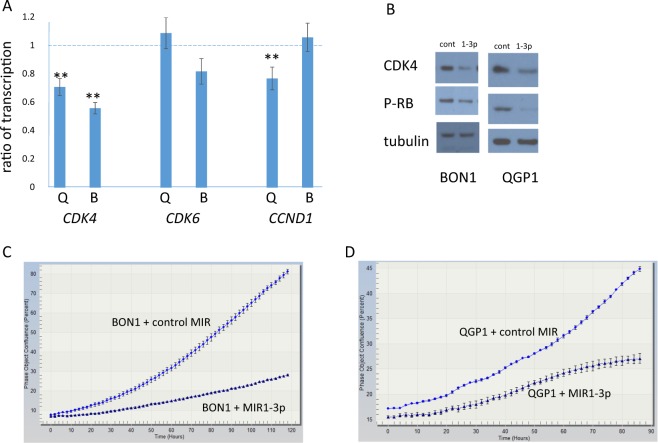
Effect of MIR1-3p on expression of *RB* pathway genes in neuroendocrine tumor cell lines. **a** Ratio of transcription refers to mRNA expression following transfection with microRNA MIR1-3p, divided by mRNA expression following transfection with a control microRNA. Values below 1 indicate repression by MIR1-3p. The assayed mRNAs were *CDK4*, *CDK6*, and *CCND1*, which were normalized to expression of *RPLPO*. Statistically significant expression differences of *CDK4* (*p* < 0.05) between cells transfected with MIR1-3p and control microRNA were determined by two-tailed *T*-test and are indicated by asterisks. Expression effects were evaluated in two neuroendocrine cell lines, QGP1 (denoted as Q) and BON1 (denoted as B). **b** QGP1 and BON1 were transfected with control microRNA or with MIR1-3p. After 48 h, protein was collected and separated by SDS–polyacrylamide gel electrophoresis. The results of Western blotting with antisera against CDK4, Phospho-RB1(807/811) and β-tubulin are shown. **c** and **d** The BON1 **c** and QGP1 **d** cell lines were transfected with MIR1-3p or with a control microRNA, and then growth was measured every 2 h using an Incucyte ZOOM live cell microscope. Quantification of plate confluence was performed using ZOOM 2016B software.

Neuroendocrine tumor cell lines were transfected with MIR1-3p. Because there are no publicly available cell lines derived from serotonin-producing ileal NETs, we performed these experiments in BON1 and QGP1, two pancreatic NET cell lines that were derived from serotonin-producing tumors^36,37^. At the mRNA level, there was no statistically significant decrease in the expression of *CDK6* or *CCND1* following MIR1-3p transfection (Fig. 2a), and *CCND2* was not assayable due to poor expression in both cell lines. However, expression of *CDK4* by both BON1 and QGP1 decreased following transfection with MIR1-3p (Fig. 2a). Transfection of MIR1-3p also decreased the amount of CDK4 protein produced by the two cell lines, as shown by Western blot (Fig. 2b). Activated CDK4 causes phosphorylation of RB1 at amino acids 807 and 811, and the amount of RB1 phosphorylated on these residues decreased in cells treated with MIR1-3p (Fig. 2b). Thus MIR1-3p decreases the activity of CDK4, which should increase the activity of RB. Transfection of MIR1-3p also decreased the rate of growth of both BON1 and QGP1 (Fig. 2c, d; see also Videos 1–4 in Supplementary material).

**Fig. 3:**
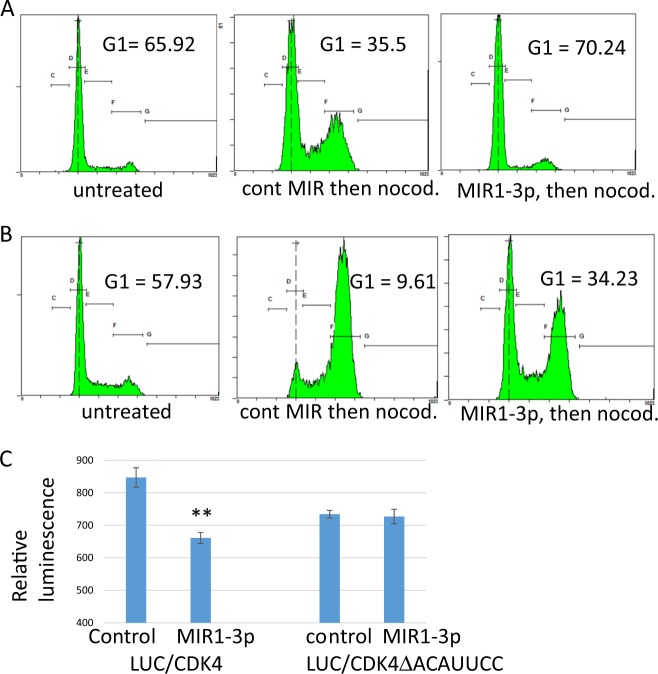
Effects of MIR1-3p on the cell cycle in neuroendocrine tumor cell lines. **a** and **b** The QGP1 **a** and BON1 **b** cell lines were transfected with MIR1-3p or with a control microRNA. 1 day later, the cells were treated with nocodazole for 16 h, followed by propidium iodide treatment and flow cytometry to determine effects on cell cycle. Untreated cells are shown in the first panel, control microRNA/nocodazole-treated cells are shown in the second panel, and MIR1-3p/nocodazole-treated cells are shown in the third panel. The percentage of cells in G1 are indicated for each treatment. **c** The cell line H1299 was transfected with control microRNA or with MIR1-3p, along with plasmids encoding either LUC/CDK4, which encodes a luciferase gene fused to the 3′ UTR of *CDK4*, or to LUC/CDK4ΔACAUUCC, which is nearly the same fusion gene except that it lacks the 7-mer sequence that is complementary to MIR1-3p. Statistical significance between control-treated and MIR1-3p-treated cells was determined by two-tailed *T*-test.

Since activated RB1 arrests growth at the G1 phase of the cell cycle^38^, we next performed cell cycle analysis on the MIR1-3p-treated cells. For this analysis, the BON1 and QGP1 cell lines were transfected with MIR1-3p or with control microRNA for 24 h, and then treated with nocodazole for another 16 h^29^. Nocodazole caused a G2 cell cycle arrest, as shown in the cells treated with control microRNA (Fig. 3a, b). But cells pre-treated with MIR1-3p were enriched in G1-phase cells, as would be expected for cells in which RB1 has been activated. Together these data indicate that MIR1-3p expression has a negative effect on growth of neuroendocrine cell lines, by decreasing the expression of CDK4.

MIR1-3p is complementary to a short segment within the 3′ UTR of *CDK4* mRNA. Fusing the *CDK4* UTR to a gene encoding firefly luciferase enabled MIR1-3p to negatively regulate luciferase expression (Fig. 3c). Conversely, following site-directed mutagenesis to remove the complementary sequence from the UTR, MIR1-3p was no longer able to regulate expression of the luciferase/CDK4 fusion gene (Fig. 3c).

A set of 57 flash-frozen human I-NETs was purchased from the Cooperative Human Tissue Network (CHTN). Patients ranged in age from 41 to 89, but 95% of the patients were 49 or older. The average age of these patients was 61. Both sexes were represented (51% male), but the samples were not racially diverse (93% Caucasian). There was no clinical data available for these patients other than the fact that 51% of patients had localized disease, and 49% of the patients had distal metastases. Distal metastases usually localized to the liver, but one patient had a metastatic neuroendocrine lesion on an ovary. Genomic DNA was prepared from these tumor samples in order to determine the copy number of the *MIR1-2* gene for each patient.

**Fig. 4:**
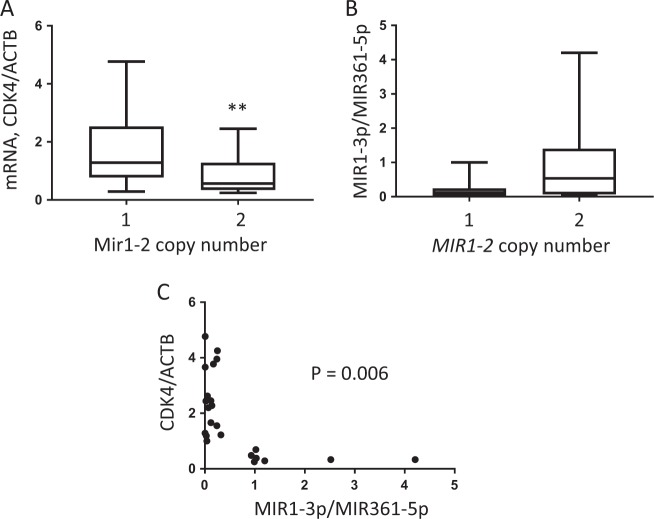
Expression of MIR1-3p and *CDK4* in human ileal neuroendocrine tumors with different copy numbers of *MIR1-2*. **a** Comparison of expression of *CDK4* mRNA by human I-NETs with loss of copy of *MIR1-2* (denoted as 1) to expression of *CDK4* by tumors with a normal copy number of *MIR1-2* (denoted as 2). *CDK4* mRNA was assayed by real-time RT-PCR and normalized to expression of a housekeeper, *ACTB*. Statistical significance was determined by Mann–Whitney analysis (*p* < 0.05). **b** Comparison of expression of MIR1-3p by human I-NETs with loss of copy of *MIR1-2* (denoted as 1) to expression by tumors with a normal copy number of *MIR1-2* (denoted as 2). MIR1-3p expression was assayed by real-time RT-PCR and normalized to expression of a housekeeper, MIR361-5p. Although there is a trend toward lower expression in tumors with loss of copy, this difference was not statistically significant by Mann–Whitney analysis (*p* = 0.12). **c** Expression of MIR1-3p and *CDK4* by individual I-NETs is plotted. The Spearman correlation coefficient was *r* = −0.556, and there was a statistically significant correlation with a *p* value of 0.006.

Expression of *CDK4* mRNA increased in tumors with loss of copy of *MIR1-2* (Fig. 4a). Conversely, expression of MIR1-3p tended to decrease in tumors with loss of copy of *MIR1-2*, although this result was not statistically significant (Fig. 4b). Plotting expression of MIR1-3p and *CDK4* mRNA revealed that these two RNAs were negatively correlated (Fig. 4c). Combined with the results from the cell line experiments, these data suggest that loss of chromosome 18 may lower expression of MIR1-3p, which can increase expression of CDK4 and cause dysregulated growth of enteroendocrine cells of the ileum.

**Fig. 5:**
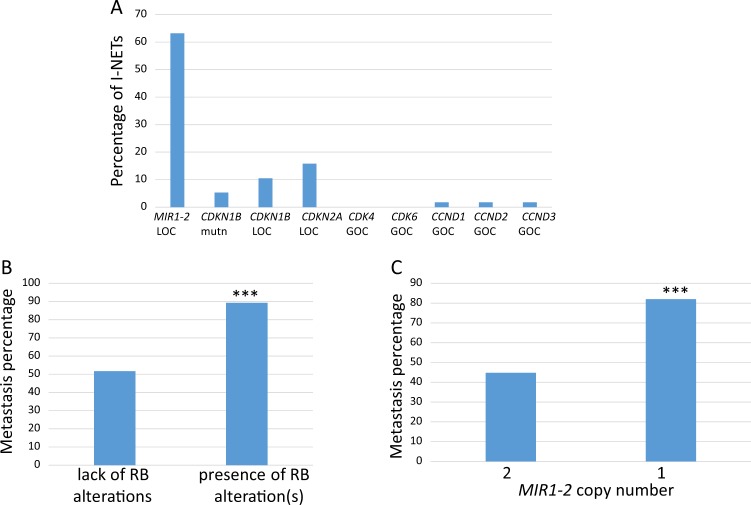
Copy numbers of *MIR1-2* and other *RB* pathway genes in metastatic or localized human ileal neuroendocrine tumors. **a** A total of 57 I-NETs were analyzed for loss of copy (LOC) of RB activators *MIR1-2*, *CDKN1B*, and *CDKN2*, for mutations in *CDKN1B*, or for gain of copy (GOC) of RB suppressors *CDK4*, *CDK6*, *CCND1*, *CCND2*, and *CCND3*. The percentage of tumors showing each of these genetic events is indicated. **b** This figure shows the incidence of metastatic disease among patients whose tumors lacked any of the genetic changes in the RB pathway shown in **a**, compared to the incidence of metastatic disease among patients whose tumors had at least one of the genetic changes in **a**. Statistical significance was determined by Fisher’s exact test (*p* < 0.01). **c** This figure shows the incidence of metastatic disease among patients whose tumors had two copies of the *MIR1-2* gene (2) compared to the incidence of metastatic disease among patients whose tumors had loss of copy of *MIR1-2* (1). Statistical significance was determined by Fisher’s exact test (*p* < 0.01).

We also assayed the tumor DNAs for mutations and copy number alterations in other genes of the RB pathway. Loss of copy of the *MIR1-2* gene was by far the most common event, occurring in 63% of tumors (Fig. 5a). *CDKN1B* mutations, and loss of copy of *CDKN1B* were uncommon. The frequencies of these alterations in *CDKN1B* within this data set were consistent with previous reports^23,25^. Loss of copy of another CDK inhibitor, *CDKN2A*, was as common as alterations in *CDKN1B* (Fig. 5a). Although amplifications of the *CDK4* and *CDK6* genes have been detected in pancreatic NETs^29^, these genes were not amplified in any of the 57 I-NET samples (Fig. 5a). There were rare amplifications of the D-cyclin genes (*CCND1*, *CCND2*, and *CCND3*); interestingly, each of these events occurred in tumors from patients with metastatic disease.

Attenuation of the RB pathway has been linked to metastasis of other tumor types^39–41^, and appears to be the case for I-NETs as well. As shown in Fig. 5b, tumors that showed copy number or sequence alterations of one or more RB pathway genes were more likely to derive from patients with metastatic disease. *MIR1-2* loss alone also correlated with metastasis, as 82% of I-NETs from patients with distal metastasis had a loss of copy of *MIR1-2*, but *MIR1-2* was lost in only 45% of patients with localized disease (Fig. 5c).

## Discussion

There is a clinical need for better treatments of metastatic I-NETs. Primary I-NETs remain difficult to detect, and at initial diagnosis most patients already have metastatic disease. Indeed I-NETs are one of the few tumor types for which there has been a recent increase in the percentage of advanced disease among newly diagnosed patients^5^. Surgical resection is the most effective treatment for I-NETs, but resection is not always possible for patients with a high metastatic burden. Pharmacologically, I-NETs do not respond to chemotherapy and are not thought to be good candidates for new immunotherapies due to a low number of tumor-specific antigens. Personalized medicine approaches for I-NETs are limited by the low number of mutations. Patients can respond to mTOR inhibitors^42^, and to somatostatin analogs such as octreotide^43^. There have been promising results from recent clinical trials in which patients with highly metastatic I-NETs were treated with toxic, radiolabeled compounds that are attached to somatostatin analogs^44,45^.

CDK4/CDK6 inhibitors are among the most exciting new anti-cancer drugs of the past 10 years, and are approved by the FDA for patients with ER+HR− breast cancer^46^. Our previous report on the incidence of CDK4 overexpression/gene amplification in pancreatic NETs, combined with the response of PNET cell lines to CDK4/CDK6 inhibitors particularly in combination with mTOR inhibitors^29^, helped lead to multiple ongoing clinical trials for neuroendocrine and breast tumors (ClinicalTrials.gov Identifiers NCT02420691, NCT03070301, and NCT02732119). But patients with I-NETs were excluded from these trials due to a lack of data about whether CDK4 is important in the biology of I-NETs. The present report strongly suggests that patients with I-NETs could benefit from treatment with CDK4/CDK6 inhibitors.

We demonstrate that MIR1-2 encodes a microRNA that negatively regulates expression of Cdk4. MIR1-2 resides on chromosome 18, whose loss is the most frequent genetic event in I-NETs, suggesting that MIR1-2 loss is a genetic driver of these tumors. Transfection of neuroendocrine tumor cell lines with the product of *MIR1-2*, MIR1-3p, decreased expression of CDK4, decreased phosphorylation of RB, prevented cell growth and caused cell cycle arrest. In patient samples, the copy number of *MIR1-2* negatively associated with MIR1-3p expression and positively associated with *CDK4* expression. Loss of copy of *MIR1-2* was particularly common in patients with advanced I-NETs. In agreement with these data, two recent studies linked a number of microRNAs with I-NET biology, including MIR1-3p, which was found in lowered abundance in patients with advanced I-NETs^34,35^. Although I-NETs are usually already metastatic at diagnosis, several of the patients in the data base had no distal metastasis, but still showed loss of copy of *MIR1-2*. These patients may be at greater risk for later development of distal metastasis.

Because of the slow growth rate of I-NETs, clinical trials can be long and costly; trials designed to test the efficacy of drugs that block cell cycle, like CDK4/6 inhibitors, also take longer than trials on drugs that cause cell apoptosis. Interestingly, in a recent study, a CDK4/6 inhibitor was tested in a set of 15 patients with various tumor types. Two of the patients showed an objective response, and one of the responding patients had an I-NET^47^. To our knowledge, this is the only literature record in which a patient with an I-NET was treated with a CDK4/6 inhibitor, and it is particularly interesting that a drug designed to prevent cell growth actually caused this I-NET to shrink. A recent report does suggest that CDK4/6 inhibitors can cause tumors to shrink by activating natural killer cells, allowing the immune system to attack tumors^48^. If CDK4/6 inhibitors can cause an objective response in a significant number of I-NETs, then perhaps shorter and less costly trials on these tumors can be designed. These trials may also have a better chance of success by stipulating that patients’ tumors show loss of copy of *MIR1-2*. In the future a personalized medicine approach, using copy number of *MIR1-2* to determine which patients should be treated with CDK4/6 inhibitors, may allow these drugs to reduce the chances of progression by some early stage patients, while also increasing the chances of objective responses of late stage patients.

The RB pathway is attenuated in many types of NETs. For instance, in previous work we showed overexpression of CDK4 in 75% of pancreatic NETs, as well as common copy number amplifications of the *CDK4* and *CDK6* genes^29^. Another type of neuroendocrine tumor, small cell lung tumors, nearly always contains *RB1* mutations^30^. *Rb1*+/− mice were initially developed to model retinoblastoma, but instead of retinoblastomas these mice developed a large variety of NETs^31,49^. RT2 mice, in which RB is inactivated by expression of SV40 T-antigen, can also develop several types of NETs (insulinomas, nonfunctioning pancreatic NETs, or duodenal NETs), depending on genetic background^9,10,50–52^. Importantly, we recently found two genetic backgrounds in which RT2 mice can also produce I-NETs^15^. For I-NETs to appear in these backgrounds, the expression of SV40 T-antigen was required, as was elevated activity of IGF2. The presence of I-NETs in these RB-inactivated mice provides further support for the importance of RB pathway attenuation in ileal neuroendocrine tumorigenesis.

## Materials and methods

### Analysis of gene expression in human I-NETs

Flash-frozen human I-NETs were obtained from the CHTN. Informed consent was obtained from CHTN and experiments were approved by the Institutional Review Boards of CHTN. Genomic DNA was prepared from 57 tumors using a Wizard kit from Promega. RNA was isolated from 42 tumors using an RNeasy kit from Qiagen. RNA was converted into cDNA using a reverse transcription reagents kit (Thermofisher), and *CDK4* was quantitated using real-time RT-PCR, then normalized to the expression of β-actin. MicroRNA was prepared from 23 frozen tumors using a miRNeasy kit from Qiagen and converted into cDNA using Taqman advanced miRNA cDNA kit (Thermofisher). MIR1-3p expression was quantitated using real-time RT-PCR, and normalized to expression of MIR361-5p, which was recommended as a housekeeping microRNA by Thermofisher. All Taqman assays were purchased from Thermofisher.

### Copy number analysis

Copy number within tumor DNA was determined by real-time RT-PCR, using an Applied Biosystems Prism 7500 and CopyCaller software, as recommended by the manufacturer. Copy number assays were purchased from Thermofisher and were as follows: *MIR1-2* (Hs06506989_cn); *CCND1* (Hs00377865_cn); *CCND2* (Hs00394283_cn); *CCND3* (Hs02982157_cn); *CDKN1B* (Hs02136152_cn); *CDKN2A* (Hs03714372_cn); *CDK4* (Hs01071103_cn); and *CDK6* (Hs00389416_cn). Copy numbers of *RNASEP* and *TERT* were used for normalization of input DNA.

### Analysis of *CDKN1B* sequence

For sequencing of *CDKN1B*, the two coding exons of *CDKN1B* were amplified from tumor DNA by PCR. PCR primers were 5′-TATCGTGAGGTCTGAAGGCC and 5′-GTTTATCAACGGTCCGCCTC (exon 2) and 5′ GCGCTTTGTTTTGTTCGGTT and 5′ AATACGCCGAAAAGCAAGCT (Exon 1). The exon 2 sequencing primer was 5′GGAGGTAGTGGGTTTTTCA and the exon 1 sequencing primer was 5′GCAAGCTAAGGTTAACAC. Sanger sequencing was performed by GeneWiz.

### Analysis of the effects of MIR1-3p expression in human neuroendocrine tumor cell lines

The QGP1 cell line was purchased from the Japan Health Sciences Foundation and was grown under 5% CO_2_ at 37 °C in RPMI media supplemented with 10% fetal bovine serum (Sigma-Aldrich). The BON1 cell line was a gift from the lab of Kjell Oberg and was grown under 5% CO_2_ at 37 °C in DMEM media supplemented with 10% fetal bovine serum. The QGP1 and BON1 cell lines were authenticated as previously described^10^. MIR1-3p (MC10617) was purchased from Thermofisher, along with a negative control (4464058). MicroRNAs were transfected using RNAifectMax (Thermofisher) as described by the manufacturer. Experiments were performed in triplicate. A total of 200,000 cells were reverse transfected with 20 pmol of microRNA in the presence of 4 μl of RNAiMAX. After 48 h, transfected cells were harvested for RNA or protein. For measurement of cell growth effects, microRNA was reverse transfected into cells, then 16 h later the wells were analyzed every 2 h using an Incucyte ZOOM live cell microscope (Essen Bioscience). Cell confluence and statistical differences were determined using ZOOM 2016B software. For measurement of cell cycle effects, microRNA was transfected for 24 h, after which cells were treated for 16 h with 100 ng/ml nocodazole (Sigma-Aldrich). Cells were harvested by trypsinization 16 h after nocodazole treatment, washed in PBS, and fixed in 70% ethanol. Propidium iodide was added for cell-cycle analysis, which was performed on a Cytomics FC500 Flow Cytometer (Beckman Coulter). Antisera against CDK4 and β-tubulin were purchased from Novus (NBP1-31308 and NB600-936, respectively). Antisera against Phospho-RB (Ser807/811) were purchased from Cell Signaling (8516S).

### Analysis of expression effects caused by MIR1-3p and the UTR of *CDK4*

To assay the effect of the 3′ UTR) of human *CDK4* mRNA on expression of firefly luciferase, the UTR was amplified from normal human genomic DNA by PCR, using primers 5′-CGCCGTGTAATTCTAGAAAGCTGCCATTTCCCTTCTG-3′ and 5′-GCCGCCCCGACTCTAGACACGCCCCGCCTAAAATC-3′. The PCR product was inserted into XbaI-digested pGL3-control vector (Promega) using an InFusion kit (Takeda). Site-directed mutagenesis using a Q5 kit (New England Biolabs) was performed to remove the luciferase/*CDK4* sequence that was complementary to MIR1-3p. Site-directed mutagenesis utilized primers 5′-CCTCCCACCTCTCCTTTT-3′ and 5′-CATTAAGGCAGCAAAGTAATC-3′. The H1299 large cell (neuroendocrine) lung cell line was a gift from the laboratory of Arnold Levine, and was plated in 12-well dishes at a concentration of 100,000 cells/well. 0.5 μg of firefly-reporter plasmid was transfected along with 20 pmol of microRNA and 2 ng of renilla-reporter plasmid pRL-TK (Promega). 1.25 μl of lipofectamine 2000 (Thermofisher) was used as a transfection reagent. Transfections were performed in triplicate for 56 h, after which the activities of firefly and Renilla luciferases were determined.

### Statistical analysis

Graphpad Prism 7.04 software was used for statistical analysis. Comparisons of cell line expression of mRNAs and luciferase were performed by two-tailed *T*-test. Tumor expression of MIR1-3p and *CDK4* were not a Gaussian distribution, and were analyzed by non-parametric Mann–Whitney test. Outliers were identified by Rout analysis. Correlation between expression of MIR1-3 and *CDK4* was evaluated by Pearson test. Comparison of metastasis percentages by tumors lacking or containing copy number changes in *MIR1-2* or in a larger set of *RB1* pathway genes was performed by two-sided Fisher’s exact test.

## Supplementary information

Supplemental Table 1

Legends for Supplemental Files

Supplemental File 1

Supplemental File 2

Supplemental File 3

Supplemental File 4
