# Supplementary material for: Loss of copy of MIR1-2 increases CDK4 expression in ileal neuroendocrine tumors
Source: Oncogenesis. 2020 Mar 20;9(3):37. doi: 10.1038/s41389-020-0221-4 (PMC7083839; doi:10.1038/s41389-020-0221-4)
Supplement: Supplementary file 1 — Supplemental Table 1 [file 41389_2020_221_MOESM1_ESM.docx]

Supplemental Table 1

**microRNA target mRNA(s) gene chromosome**

MIR29-3p *CDK6*, *CCND2* *MIR29B2* 1

MIR34-5p *CDK6*, *CCND1* *MIR34A* 1

MIR101-3p.2 *CDK6* *MIR101-1* 1

MIR135-5p *CCND2* *MIR135B* 1

MIR137 *CDK6* *MIR137* 1

MIR190-5p *CCND2* *MIR190B* 1

MIR200a-3p *CDK6*, *CCND2* *MIR200A* 1

LET7-5p *CCND1*, *CDK6*, *CCND2* *LIN28A* 1

MIR10-5p *CDK6* *MIR10b* 2

MIR26-5p *CCND2*, *CDK6* *MIR26B* 2

MIR153-3p *CCND2* *MIR153-1* 2

MIR375 *CCND2* *MIR375* 2

MIR15-5p *CDK4, CCND1, CCND2, CCND3, CDK6* *MIR15b* 3

MIR16-5p *CDK4, CCND1, CCND2, CCND3, CDK6* *MIR16-2* 3

MIR26-5p *CCND2, CDK6*  *MIR26A1* 3

MIR135-5p *CCND2*  *MIR135A1* 3

MIR138-5p *CCND3, CDK6* *MIR138-1* 3

MIR191-5p *CDK6* *MIR191* 3

MIR302c-3p.2 *CCND1, CCND2*  *MIR302c* 4

MIR302-3p *CCND1, CCND2* *MIR302A* 4

MIR103-3p *CDK6* *MIR103A1* 5

MIR145-5p *CCND2, CDK6*  *MIR145* 5

MIR449-5p *CCND1, CDK6* *MIR449A* 5

MIR1271-5p *CCND2, CCND3*  *MIR1271* 5

MIR206 *CCND1, CDK4, CCND2, CDK6* *MIR206* 6

MIR29-3p *CDK6, CCND2*  *MIR29A* 7

MIR29-3p *CDK6, CCND2* *MIR29b1* 7

MIR93-5p *CCND1, CDK6, CCND2* *MIR93* 7

MIR96-5p *CCND2, CCND3*  *MIR96* 7

MIR106-5p *CCND1, CDK6, CCND2* *MIR106B* 7

MIR129-5p *CDK6* *MIR129-1* 7

MIR148-3p *CDK6* *MIR148A* 7

MIR153-3p *CCND2* *MIR153-2* 7

MIR182-5p *CCND2, CCND3, CDK6* *MIR182* 7

MIR183-5p.1 *CCND2* *MIR183* 7

MIR196-5p *CCND2* *MIR196B* 7

MIR590-5p *CDK6* *MIR590* 7

MIR124-3p.1 *CCND2, CCND3* *MIR124-1* 8

**microRNA target mRNA(s) gene chromosome**

MIR124-3p.2 *CDK4, CDK6 MIR124-2* 8

MIR383-5p.1 *CCND2 MIR383*  8

MIR27-3p *CDK6 MIR27B*  9

MIR101-3p.2 *CDK6 MIR101-2* 9

MIR204-5p *CCND2 MIR204*  9

MIR107 *CDK6 MIR107* 10

MIR34-5p *CDK6, CCND1 MIR34B*  11

MIR129-5p *CDK6 MIR129-2* 11

MIR130-3p *CCND3 MIR130A*  11

MIR139-5p *CCND2, CDK6 MIR139*  11

MIR302-3p *CCND1, CCND2 MIR302*  11

MIR26-5p *CCND2, CDK6 MIR26A2*  12

MIR135-5p *CCND2 MIR135A2* 12

MIR141-3p *CDK6, CCND2 MIR141*  12

MIR148-3p *CDK6 MIR148*  12

MIR196-5p *CCND2 MIR196A2*  12

MIR15-5p *CDK4, CCND1, CCND2, CCND3, CDK6 MIR15a*  13

MIR16-5p *CDK4, CCND1, CCND2, CCND3, CDK6 MIR16-1* 13

MIR17-5p *CCND1, CCND2, Cdk6 MIR17* 13

MIR18-5p *CCND2 MIR18A*  13

MIR19-3p *CCND1, CCND2 MIR19A*  13

MIR20-5p *CCND1, CCND2, Cdk6 MIR20A*  13

MIR190-5p *CCND2 MIR190A* 15

MIR211-5p *CCND2 MIR211*  15

MIR138-5p *CCND3, CDK6 MIR138-2* 16

MIR140-3p.2 *CDK6 MIR140*  16

MIR193-3p *CCND1 MIR193B* 16

MIR10-5p *CDK6 MIR10A*  17

MIR21-5p *CDK6 MIR21*  17

MIR33-5p *CDK6 MIR33B*  17

MIR142-3p.2 *CCND1 MIR142*  17

MIR142-5p *CDK6, CCND1 MIR142*  17

MIR152-3p *CDK6 MIR152*  17

MIR193-3p *CCND1 MIR193A* 17

MIR195-5p *CDK4, CDK6, CCND1,CCND2, CCND3 MIR195*  17

MIR196-5p *CCND2 MIR196A1* 17

MIR212-5p *CDK6, CCND3 MIR212*  17

MIR301-3p *CCND3 MIR301A* 17

MIR338-3p *CDK4 MIR338*  17

**microRNA target mRNA(s) gene chromosome**

MIR454-3p *CCND3 MIR454*  17

MIR497-5p CDK4, CDK6, CCND1, CCND2, CCND3 MIR497 17

MIR1-3p *CDK4, CDK6, CCND1, CCND2 MIR1-2* 18

MIR23-3p *CCND1 MIR23A*  19

MIR27-3p *CDK6 MIR27A*  19

MIR150-5p *CCND2 MIR150*  19

MIR372-3p *CCND1, CCND2 MIR372*  19

MIR373-3p *CCND1, CCND2 MIR373*  19

MIR519-3p *CCND1, Cdk6, CCND2 MIR519A1* 19

MIR520-3p *CCND1, CCND2 MIR520A* 19

MIR1-3p *CDK4, CDK6, CCND1, CCND2 Mir1-1*  20

MIR103-3p *CDK6 MIR103A2* 20

MIR124-3p.1 *CCND2, CCND3 MIR124-3* 20

MIR124-3p.2 *CDK4, CDK6 MIR124-3* 20

MIR499a-5p *CDK6 MIR499A* 20

MIR802 *CCND2 MIR802*  21

MIR33-5p *CDK6 MIR33A*  22

MIR130-3p *CCND3 MIR130B*  22

MIR301-3p *CCND3 MIR301B* 22

MIR18-5p *CCND2 MIR18B* X

MIR19-3p *CCND1, CCND2 MIR19B2*  X

MIR20-5p *CCND1, CCND2, CDK6 MIR20B*  X

MIR23-3p *CCND1 MIR23C*  X

MIR98-5p *CDK6, CCND1, CCND2 MIR98*  X

MIR106-5p *CCND1, CDK6, CCND2 MIR106A* X

MIR424-5p *CCND1, CCND2, CCND3, CDK4, CDK6 MIR424* X

MIR506-3p *CDK4, CDK6, CCND2 MIR506* X
