# Supplementary material for: Loss of copy of MIR1-2 increases CDK4 expression in ileal neuroendocrine tumors
Source: Oncogenesis. 2020 Mar 20;9(3):37. doi: 10.1038/s41389-020-0221-4 (PMC7083839; doi:10.1038/s41389-020-0221-4)
Supplement: Supplementary file 2 — Legends for Supplemental Files [file 41389_2020_221_MOESM2_ESM.docx]

**Supplemental Videos**

**Videos 1 and 2.** The BON1 cell line was treated with control microRNA (Video 1) or with MIR1-3p (Video 2) for 16 hours, then analyzed for growth using an Incucyte ZOOM live cell microscope. Photos were taken every two hours. Photos from a representative section of the two plates are shown in these two videos, and a summary of data extracted from multiple positions on the two plates is shown in Figure 2C.

**Videos 3 and 4.** The QGP1 cell line was treated with control microRNA (Video 3) or with MIR1-3p (Video 4) for 16 hours, then analyzed for growth using an Incucyte ZOOM live cell microscope. Time lapse photos from a representative section of the two plates are shown in these two videos, while a summary of data extracted from multiple positions on the two plates is shown in Figure 2D.
